# Diagnostic Value of CEUS Prompting Liver Biopsy: Histopathological Correlation of Hepatic Lesions with Ambiguous Imaging Characteristics

**DOI:** 10.3390/diagnostics11010035

**Published:** 2020-12-27

**Authors:** Thomas Geyer, Dirk-André Clevert, Sonja Schwarz, Paul Reidler, Sebastian Gassenmaier, Thomas Knösel, Johannes Rübenthaler, Vincent Schwarze, Marco Armbruster

**Affiliations:** 1 Department of Radiology, Ludwig-Maximilians-University, 81377 Munich, Germany; dirk.clevert@med.uni-muenchen.de (D.-A.C.); sonjaschwarz128@gmail.com (S.S.); paul.reidler@med.uni-muenchen.de (P.R.); johannes.ruebenthaler@med.uni-muenchen.de (J.R.); vincent.schwarze@med.uni-muenchen.de (V.S.); marco.armbruster@med.uni-muenchen.de (M.A.); 2 Department of Diagnostic and Interventional Radiology, University Hospital Tuebingen, 72076 Tuebingen, Germany; sebastian.gassenmaier@med.uni-tuebingen.de; 3 Institute of Pathology, Ludwig-Maximilians-University, 81377 Munich, Germany; Thomas.knoesel@med.uni-muenchen.de

**Keywords:** CEUS, contrast-enhanced ultrasound, liver, biopsy, hepatocellular carcinoma

## Abstract

Background: Contrast-enhanced ultrasound (CEUS) allows for dynamic analysis of vascularization patterns of unclear hepatic lesions. Our study aimed to evaluate the diagnostic performance of CEUS for further characterizing suspicious liver lesions by comparing findings from CEUS examinations with corresponding histopathology. Methods: Between 2005 and 2016, 160 patients with unclear liver lesions underwent CEUS followed by liver biopsy. All examinations were performed by an experienced consultant radiologist (EFSUMB Level 3) and included native B-mode ultrasound, Color Doppler, and CEUS. A second-generation blood pool contrast agent was applied for CEUS. Results: CEUS was successfully performed in all patients without occurrence of any adverse side effects. CEUS showed a sensitivity of 94.5%, a specificity of 70.6%, a true positive rate of 87.3%, and a true negative rate of 85.7% compared to histopathological results as the reference standard. Conclusions: CEUS represents a safe imaging modality with a high diagnostic accuracy in assessing both—benign and malignant—liver lesions compared to corresponding histopathological results.

## 1. Introduction

Hepatocellular carcinoma (HCC) depicts the most common primary hepatic malignancy and the second most lethal cancer entity [1,2]. For HCC development, there is a strong predilection in men. Major risk factors comprise chronic hepatitis B and C infections, non-alcoholic fatty liver disease (NAFLD), alcoholic liver disease (ALD), or congenital disorders like hemochromatosis [3]. Approximately 90% of HCC develop in underlying liver cirrhosis [4].

Imaging of patients with suspected HCC is of utmost importance and diagnosis is predominantly based on CT or MRI, while biopsy is only performed in indeterminate cases or to define immunologic treatment options [5,6]. The first line imaging tool to screen for HCC lesions in high-risk patients is conventional ultrasound, comprising native B-mode and Color Doppler.

Contrast-enhanced ultrasound (CEUS) is a well-established, non-invasive method for efficient and cost-effective diagnostics [7,8,9,10,11,12,13]. The advantages of CEUS are based on its non-invasive nature, its direct availability and repeatability. Furthermore, the contrast agents applied in the context of CEUS present excellent safety profiles which are far superior compared to iodine- or gadolinium-based contrast media in case of computed tomography (CT) and magnetic resonance imaging (MRI), respectively. Ultrasound contrast agents do not affect the renal or thyroid function, and allergic reactions occur less frequently [14]. Therefore, CEUS is increasingly applied in various medical fields and provides substantial added diagnostic value for differentiating hepatic lesions [7,8,9,10,11,12,15,16,17,18].

Contrast-enhanced ultrasound has been established as the third imaging modality in the definite diagnosis of HCC and was affirmed in the 2017 iteration of the Liver Imaging Reporting and Data System (CEUS LI-RADS) [19]. Besides, a variety of further benign or malignant hepatic lesions can be identified by applying CEUS [20]. However, the inherent dependency on operator experience makes the use of CEUS outside of high-volume centers controversial [21]. Still, it is among the recommended procedures to define HCC therapy solely according to CEUS. This is supported by reports of high sensitivity and specificity in studies with a focus on patients with cirrhosis [22,23,24,25,26].

In contrast to this extensive research on HCC, the real-world scenario of indeterminate, yet suspicious focal lesions requiring histopathological validation remains unexplored [17]. Therefore, the role of CEUS prompting liver biopsy in this setting needs further investigation. This gains importance, as specific guidelines are missing and peri- and postprocedural risks of biopsy like hemorrhage or tumor seeding need to be considered for each patient individually [21].

This study aimed to compare findings from CEUS examinations of suspicious liver lesions with corresponding histopatholoy as the reference standard and to evaluate the diagnostic performance of CEUS for further characterizing unclear hepatic lesions.

## 2. Materials and Methods

This retrospective single-center study was approved by the local institutional review board (Ethics Committee, Medical Faculty, Ludwig-Maximilians-University Munich; 17-087; date of approval: 14 March 2017). Informed consent was waived. All contributing authors followed the principles of the Declaration of Helsinki/Edinburgh 2002.

First of all, a database of all CEUS examinations between 2005 and 2016 with suspicion of a malignant liver lesion which were all performed by a single skilled radiologist with professional experience since 2000 (EFSUMB Level 3) was established. All included patients either had unclear liver lesions which had been detected using other imaging modalities and required further assessment by CEUS for definitive characterization, and/or suffered from conditions such as liver cirrhosis or malignant extrahepatic tumors (e.g., breast, lung, colon) and underwent CEUS in order to rule out malignant hepatic lesions. All CEUS examinations were performed with up-to-date high-end ultrasound devices (Siemens ACUSON Sequoia, S2000 and S3000, Mountain View, CA, USA; Philips EPIQ 7, Seattle, WA, USA) with adequate scanning protocols available at the date of the study corresponding to previous studies [18,27,28,29]. All transducers were suitable for abdominal imaging with a frequency ranging from 1.0 to 9.0 MHz. The Siemens ultrasound systems were used with a C4-1 and C6-1 HD probe, whereas the Philips ultrasound system required a C9-2 probe. All systems were programmed with a low mechanical index (<0.2) to prevent early microbubble destruction.

In all patients, we used 1.4–2.0 mL of a second-generation blood pool contrast agent (SonoVue^®^, Bracco, Milan, Italy) that was administered via a 20–22 gauge needle in an antecubital vein by a bolus injection followed by a saline flush of 5–10 mL. Following the contrast agent injection, cine loops of the CEUS study were acquired and saved in the picture archiving and communication system (PACS) of our institution. Mean examination time ranged between 3–5 min for the complete study.

**Figure 1:**
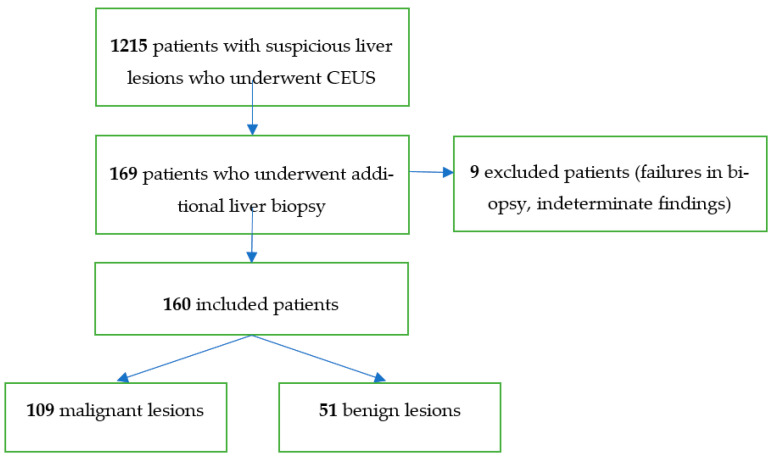
Flowchart illustrating the included patients who underwent CEUS followed by liver biopsy.

In total, 1215 patients could be included. In a second step, all patients without histopathological assessment of the suspicious lesions were excluded resulting in 169 remaining patients. Nine patients had to be excluded due to failures in biopsy and indeterminate pathologic findings so that the final study group consisted of 160 patients (Figure 1). All sonographic reports were compared with the histopathological reports regarding the classification of the suspicious lesion into malignant or benign.

Proprietary software was used for the statistical analysis (IBM SPSS Statistics, Version 23, Armonk, NY, USA; Microsoft Excel 2016, Redmond, WA, USA; GraphPad Prism, Version 7.04 for Windows, GraphPad Software, La Jolla, CA, USA). The sensitivity and specificity were calculated using the histopathological results as the reference standard. We calculated the true positive rate by dividing the number of patients which CEUS classified as malignant by the number of patients which were classified as malignant in histopathology, and the true negative rate by dividing the number of patients which CEUS classified as benign by the number of patients which were classified as benign in histopathology.

## 3. Results

In all included 160 patients, CEUS examinations were successfully conducted without occurrence of any adverse side effects. The mean age of the patients was 59 years (range: 20–83 years) with a male predominance (97 men, 63 women; ratio: 1.5:1). The mean liver lesion size was 2.6 cm (range: 0.5–8.7 cm). Image quality was sufficient in each case. The histopathological assessment was definitive in all cases.

The histopathological results are illustrated in Table 1. Most suspicious lesions were classified as metastases (*n* = 59). Primary tumor entities were colorectal cancer (*n* = 14), neuroendocrine tumors (*n* = 10), pancreatic cancer (*n* = 7), malignant melanoma (*n* = 5), breast cancer (*n* = 5), lung cancer (*n* = 5), ovarian cancer (*n* = 4), prostate cancer (*n* = 2), renal cell carcinoma (*n* = 2), leiomyosarcoma (*n* = 2), duodenal cancer (*n* = 1), mandibular cancer (*n* = 1), and cervical cancer (*n* = 1).

38 lesions were classified as HCC with 9 well differentiated, 17 moderately differentiated, and 12 poorly differentiated lesions.

In 43 cases pathological, but benign changes were diagnosed. In four cases, histopathology revealed histological features of hepatitis without patterns of malignancy. Two of these patients suffered from chronic hepatitis C and two patients from autoimmune hepatitis. In ten cases, fibrotic changes without patterns of malignancy were described, including four patients with mild liver fibrosis, two patients with moderate fibrosis, and four patients with advances fibrotic changes of the liver parenchyma. Three cases were summarized to “other reactive changes” in Table 1. In these cases, histopathological assessment revealed minor nonspecific changes of the liver parenchyma which might have been caused by medication, intoxication, or inflammation. In thirteen cases which are classified as “inconspicuous” in Table 1, no pathological changes of the liver parenchyma were revealed in histopathological assessment.

**Figure 2:**
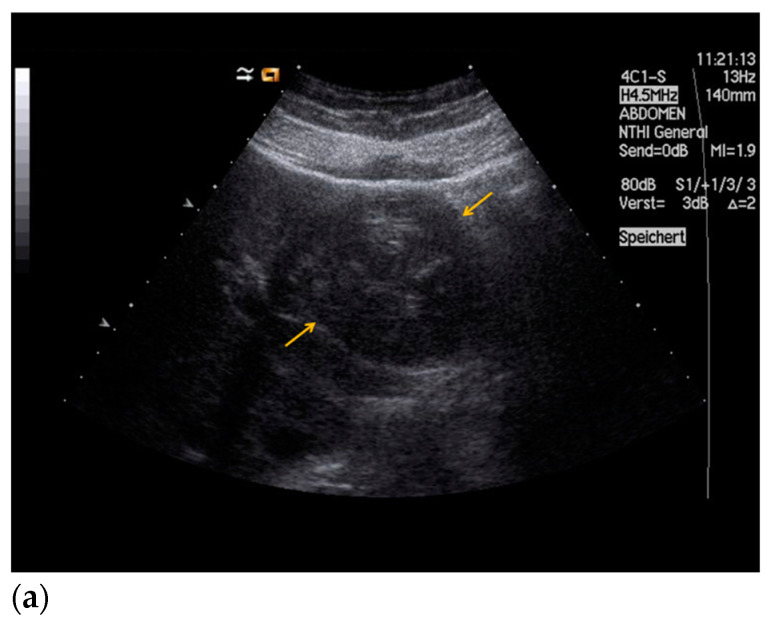

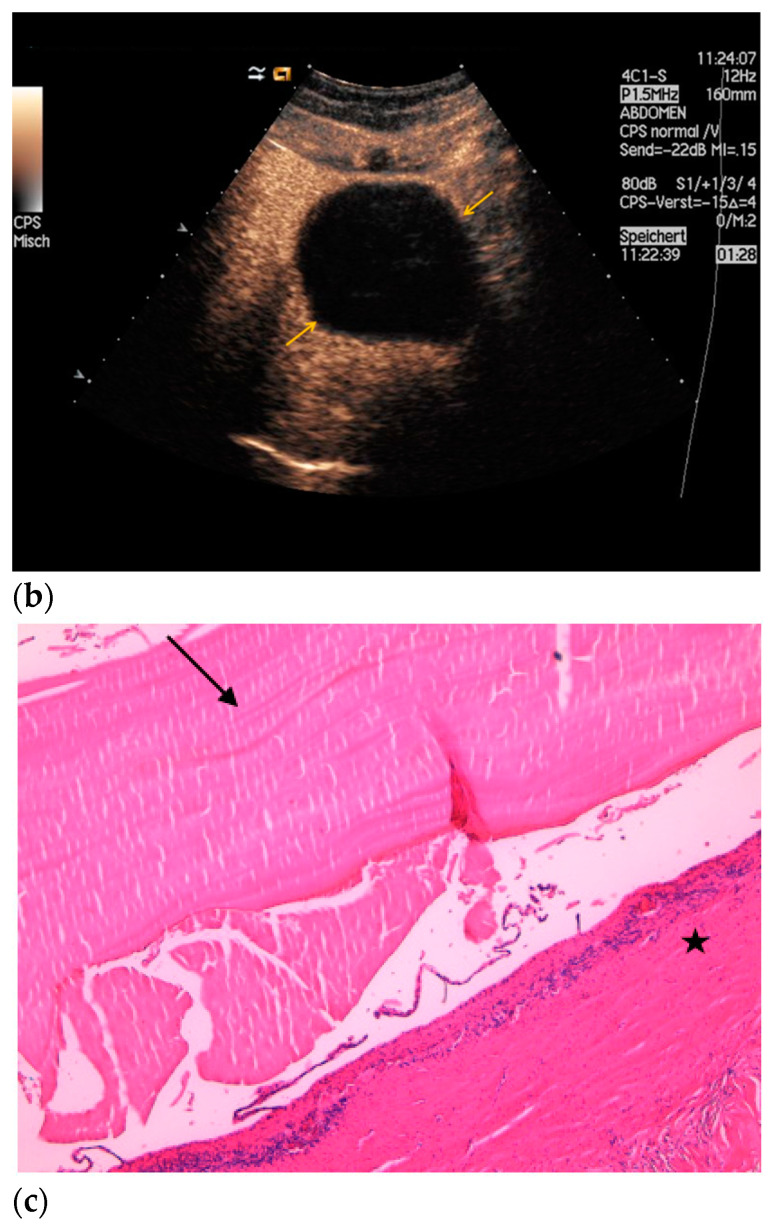
34-year-old patient with an echinococcal cyst in liver segments II/III. (**a**) Native B-mode ultrasound reveals an inhomogeneous cystic lesion in the left liver lobe (yellow arrows). (**b**) In contrast-enhanced ultrasound, no enhancement of the cystic lesion is detected (yellow arrows, portal venous phase). Sonomorphological differentials included haemorrhagic cyst or cystic echinococcosis. The sonomorphological appearance in combination with positive serological findings confirmed the diagnosis of cystic echinococcosis. After interdisciplinary evaluation, the patient underwent laparoscopic resection of the liver segments II and III. (**c**) Hematoxylin and Eosin staining of the echinococcal cyst, 50× magnification. The fibrous tissue (pericyst, black star) surrounding the cuticula (black arrow) is shown.

**Figure 3:**
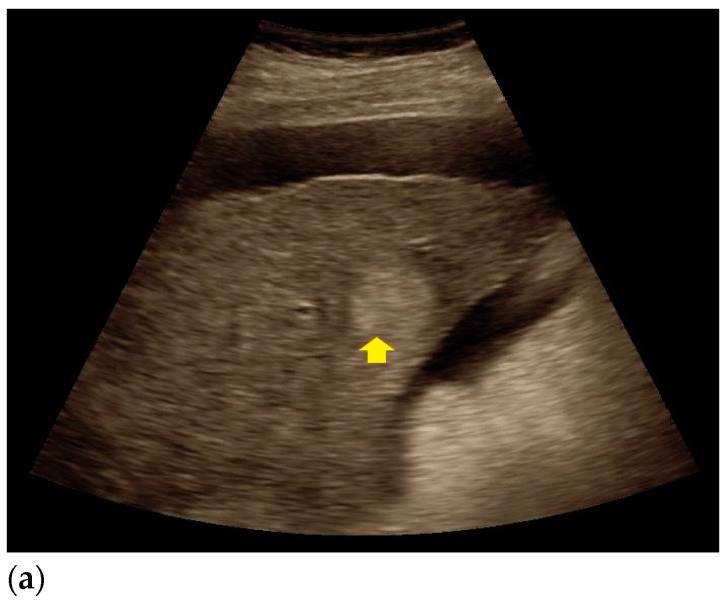

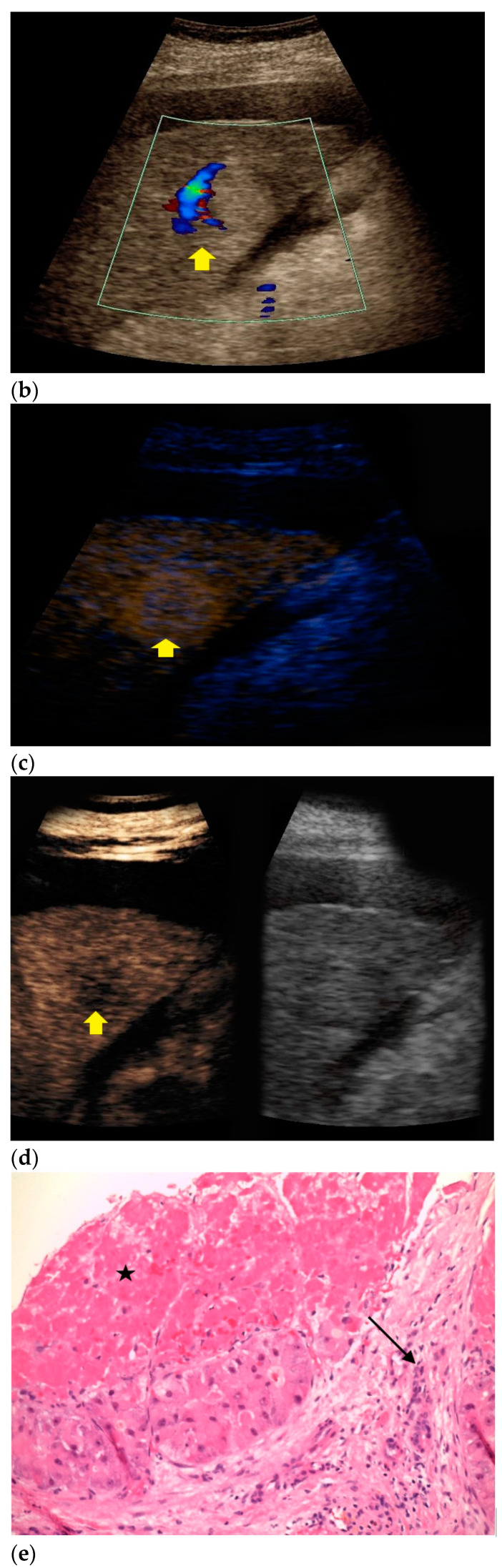
Sonomorphological features and histopathological assessment of poorly differentiated hepatocellular carcinoma (HCC). (**a**) Homogeneous, hyperechoic liver lesion in native B-mode (yellow arrow). (**b**) The lesion shows peripheral hypervascularization in Doppler mode (yellow arrow). (**c**) In CEUS, peripheral rim contrast enhancement was detected in the arterial phase (yellow arrow). (**d**) In the late venous phase, the lesion showed typical rapid wash-out (yellow arrow), thus indicating HCC. (**e**) Hematoxylin and Eosin staining of the suspicious lesion, 20× magnification. Poorly differentiated hepatocellular carcinoma with concomitant necrosis (black star) and fibrous septa (black arrow) is shown.

**Figure 4:**
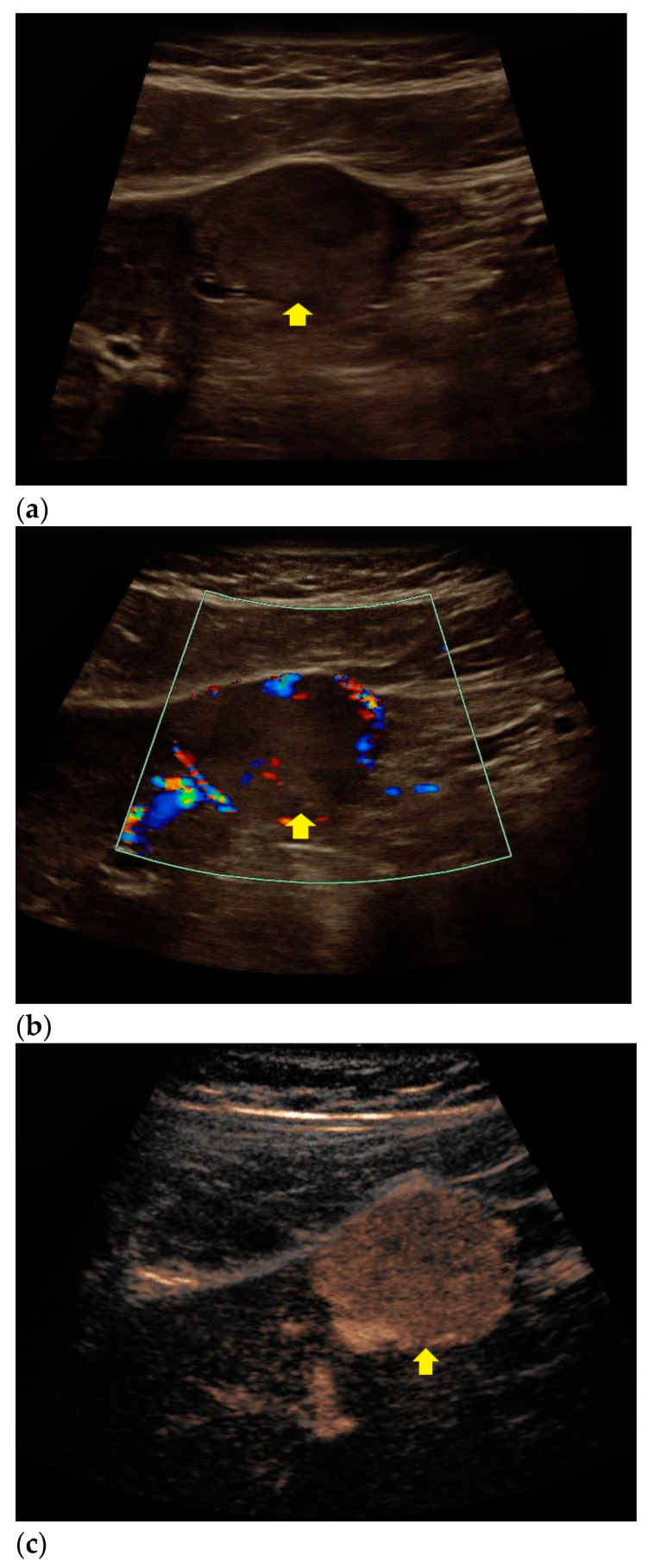

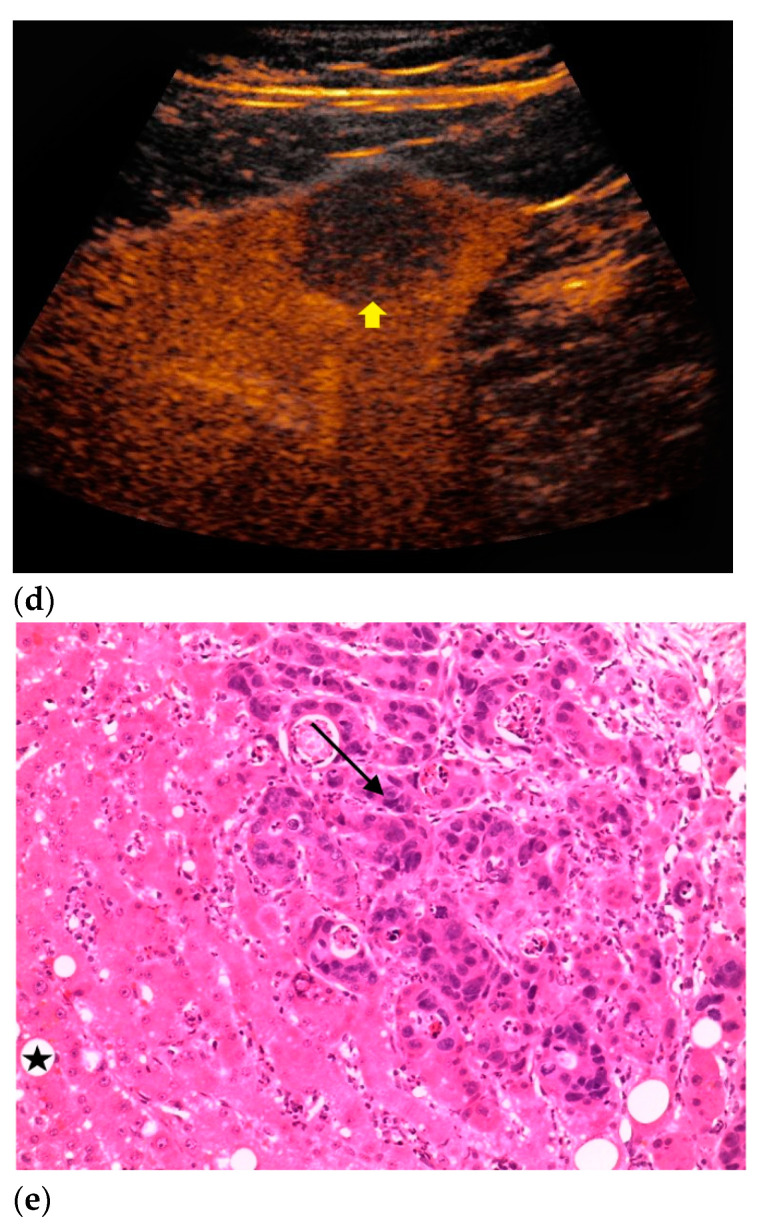
Sonomorphological features of a suspicious liver lesion in CEUS. Subsequent biopsy with histopathological assessment revealed colorectal cancer metastasis. (**a**) An inhomogeneous, slightly hyperechoic liver lesion is visualized in native B-mode (yellow arrow). (**b**) The lesion appears hypervascularized in Doppler mode (yellow arrow). (**c**) In CEUS, rapid and homogeneous contrast enhancement was registered in the arterial phase (yellow arrow). (**d**) In the venous phase, the lesion showed homogeneous wash-out compared to the liver parenchyma (yellow arrow). (**e**) Hematoxylin and Eosin staining of the lesion, 20× magnification. Colorectal adenocarcinoma metastasis is shown on the right (black arrow), normal liver tissue is shown on the left with concomitant steatosis (black star).

Findings from CEUS and histopathological assessment in patients with echinococcal cyst, HCC, and liver metastases are shown in Figure 2, Figure 3 and Figure 4.

**Figure 5:**
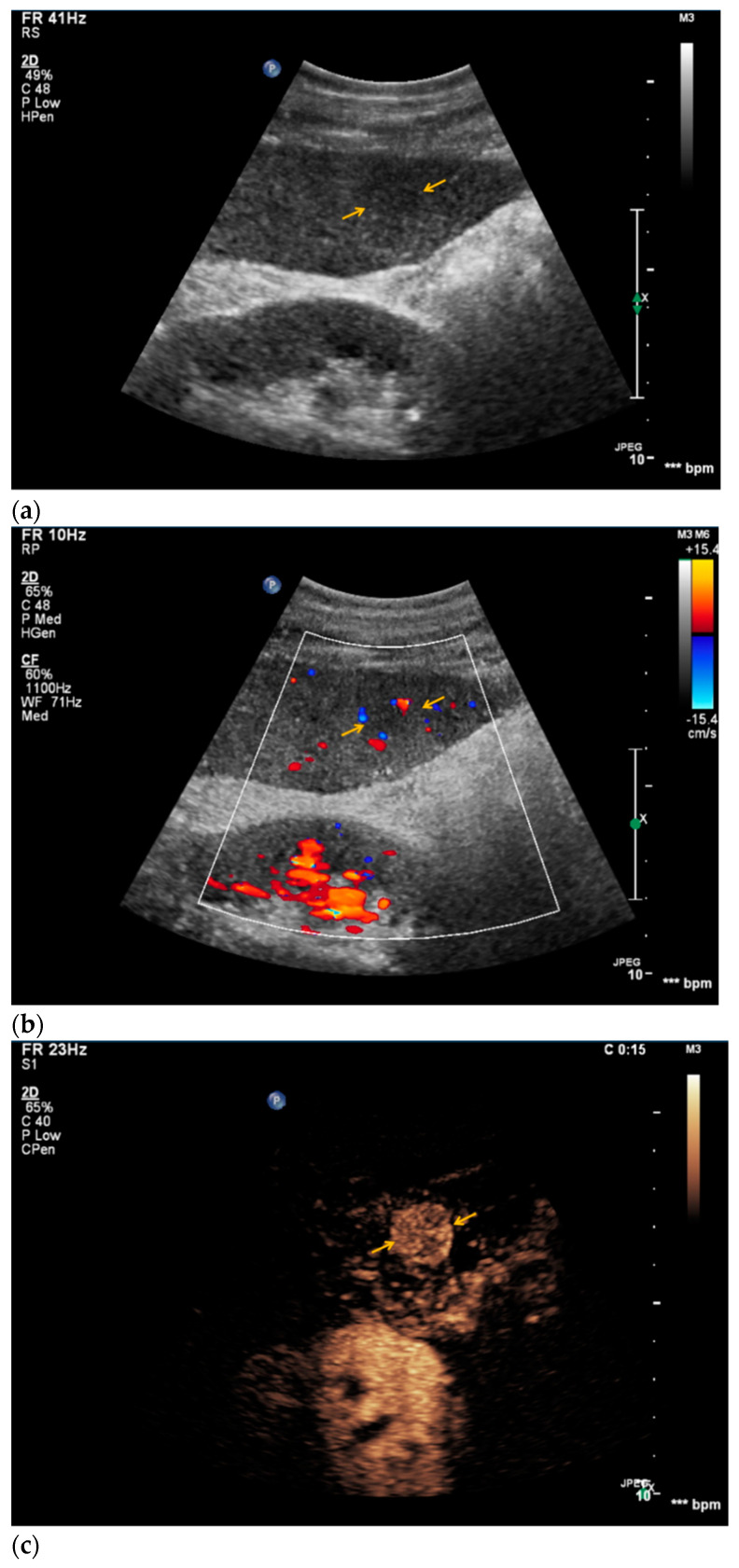

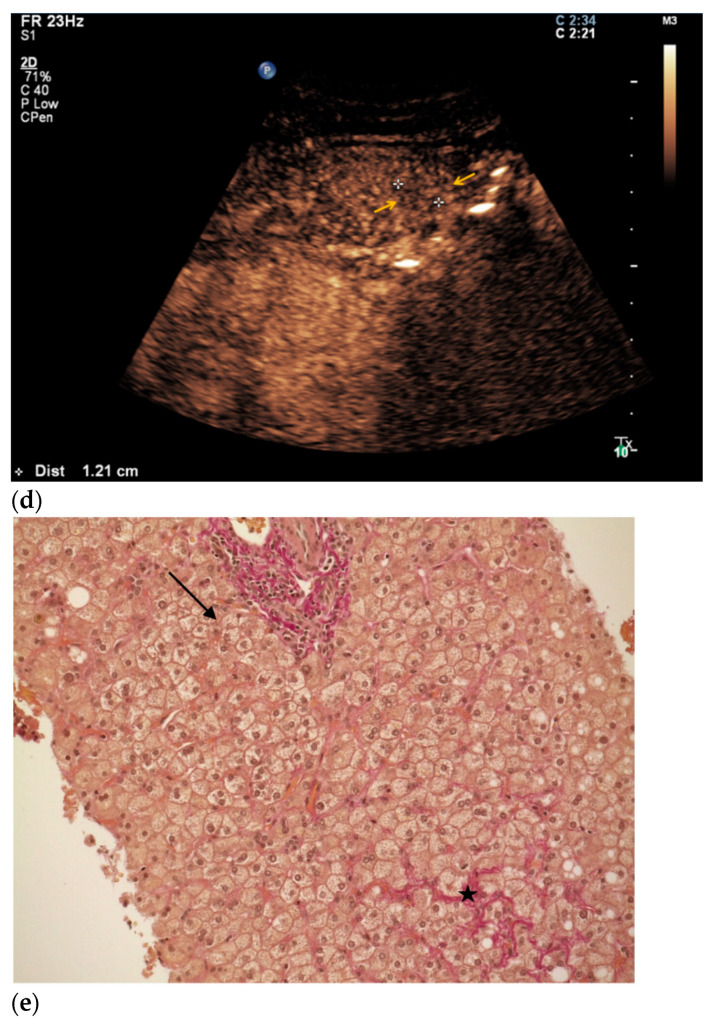
Sonomorphological features of a suspicious lesion in the right liver lobe. Subsequent histopathological assessment revealed liver fibrosis. (**a**) A slightly hypoechoic lesion is visualized in liver segment VI in native B-mode (yellow arrows). (**b**) The lesion appears hypervascularized in Doppler mode (yellow arrows). (**c**) In CEUS, rapid contrast enhancement was visualized in the arterial phase (yellow arrows). (**d**) The lesion showed only slight wash-out compared to the liver parenchyma in the venous phase (yellow arrows). Subsequent biopsy was performed due to the atypical sonomorphological appearance of the lesion. (**e**) Elastica van Gieson staining of the lesion, 20× magnification. Liver parenchyma with thin (black star) and broad (black arrow) fibrous septa is shown. No patterns of malignant neoplasms were detected.

Regarding the classification of malignant lesions, CEUS showed a sensitivity of 94.5%, a specificity of 70.6%, a true positive rate of 87.3%, and a true negative rate of 85.7% compared to histopathology as the reference standard. In 7 cases, CEUS classified lesions as malignant that revealed to be fibrotic changes in the histopathological analysis. Results from CEUS and subsequent histopathological assessment of one of these patients are shown in Figure 5. In two lesions, no pathologic changes were observed in the histopathological analysis after CEUS suspected malignancy. In three cases, cirrhotic changes were misinterpreted as malignant changes. Further lesions that CEUS misinterpreted as malignant were diagnosed as a hepatocellular adenoma, as reactive changes in the liver, and as a liver abscess.

In four cases CEUS characterized lesions as benign that were finally diagnosed as HCC in histopathology (lesion sizes: 0.8 cm, 2.0 cm, 3.4 cm, 6.2 cm). In one case CEUS described a lesion as an hepatic abscess that was histopathologically classified as a pancreatic cancer metastasis (lesion size: 6.9 cm). One lesion that CEUS characterized as benign revealed to be a metastasis of a well-differentiated neuroendocrine tumor originated in the ileum (lesion size: 2.5 cm). Diagnostic characteristics and details on misclassifications are displayed in Table 2 and Table 3.

## 4. Discussion

Contrast-enhanced ultrasound is a frequently used imaging modality that allows for characterizing unclear hepatic lesions by dynamically assessing lesional and parenchymal microperfusion. The findings from the present study demonstrate that CEUS depicts a valid and reliable method for characterizing suspicious liver lesions of unclear origin into benign or malignant.

Our study is in line with previous investigations regarding the decision between benign and malignant liver lesions using CEUS [18,30,31,32,33]. Our data clearly show that CEUS has high diagnostic accuracy in assessing both benign and malignant hepatic lesions and thereby represents a powerful diagnostic tool that can be used as a first means for assessing unclear liver lesions. Previously, it was already shown that CEUS provides high diagnostic accuracy for analyzing HCC lesions, hemangioma, and focal nodular hyperplasia [20,30]. Our results indicate that the application of CEUS may not only be useful in diagnosing these entities but may also enable the diagnosis of a variety of further liver diseases. Nevertheless, further trials are necessary to investigate if CEUS may help to define the origin of liver metastases. Still, CEUS is not able to replace CT and MRI for adequate tumor staging.

Amongst the liver lesions which CEUS wrongly classified as malignant, histopathology most frequently revealed fibrotic lesions (*n* = 7) and cirrhotic lesions (*n* = 3). In patients with advanced liver cirrhosis, the destruction of the hepatic parenchyma may result in confluent liver fibrosis which is known to show varying characteristics in dynamic contrast-enhanced imaging. Confluent liver fibrosis may show hyperenhancement in the arterial phase and thereby mimic the enhancement characteristics of HCC, thus prompting liver biopsy [34]. In two cases, liver biopsy and histopathological assessment revealed hepatic cysts. However, these lesions had shown suspicious sonomorphological features in CEUS (solid components with early enhancement and late wash-out). Therefore, biopsy was performed in order to rule out malignancy.

In four cases, CEUS misclassified hepatic lesions as benign which revealed as HCC in the histopathological assessment. In one of these cases, the patient underwent hepatectomy due to liver cirrhosis and a solitary small well-differentiated HCC lesion (0.8 cm) was detected in the enlarged left liver lobe in the histopathological analysis. The diagnostic accuracy of CEUS might have been limited by the hepatomegaly, the advanced cirrhotic changes of the liver parenchyma, and the small lesion size. Two further well-differentiated HCC lesions were wrongly classified as benign due to their atypical contrast enhancement patterns with absence of wash-out in the late phase. Typically, HCC lesions are characterized by early arterial contrast enhancement and portal venous wash-out in contrast-enhanced imaging. However, it is known that well-differentiated HCCs generally show atypical contrast enhancement characteristics in CEUS more frequently [35]. In one case, CEUS misdiagnosed an HCC lesion as a hepatic abscess. In addition, one lesion which CEUS initially classified as malignant revealed to be an abscess. Liver abscesses are known to show heterogeneous enhancement patterns and can therefore complicate safe differentiation from HCC in some cases [36]. Therefore, the patient’s clinical presentation including symptoms like fever or elevated inflammatory blood markers should also be considered for allowing safe discrimination between HCC and liver abscesses.

One lesion which CEUS misdiagnosed as benign was eventually revealed as neuroendocrine metastasis by histopathology. This is in line with previously published data indicating that CEUS is less sensitive for characterizing neuroendocrine metastases compared to metastases of different origin [37]. Neuroendocrine metastases tend to show prolonged arterial contrast enhancement due to their hypervascularization which may overlap the lesions’ hypoenhancement especially in the early venous phase.

Up to recently, clinical studies investigating CEUS for a plethora of indications have considerably increased. Several recent studies could show that CEUS does not only allow for precise liver imaging but is also feasible for visualizing the kidney, gallbladder, pancreas, or the vasculature [8,9,10,29,38].

Moreover, it was recently demonstrated that the combination of CEUS with previously acquired CT or MRI data in the context of advanced Fusion Imaging could help to decipher initially as indeterminate classified hepatic lesions [39].

The advantages of CEUS are primarily based upon its non-ionizing nature that is especially relevant in patients of younger age. Low financial costs, and its easy accessibility and repeatability are also expedient. Sonographic contrast agents bear only few risks and can be applied regardless of renal and thyroid impairment. Several authors previously described the safe use of CEUS in pediatric patients which therefore was recently approved by the Food and Drug Administration [40]. Furthermore, recent studies showed that CEUS also depicts a safe imaging tool for assessing hepatic lesions during pregnancy, thereby preventing potential risks of additional use of gadolinium-based contrast agents in MRI or ionizing radiation in CT [31,41]. However, CEUS provides only limited information in patients with high body mass index or bowel gas overlay. Another important limitation is the dependence on the skills and experience of the sonographer.

There are several limitations to this study. Firstly, this was a single-center study that was performed retrospectively. Secondly, only one single radiologist (however, with long-lasting experience since 2000 and EFSUMB Level 3) performed all sonographic procedures. Thirdly, several different ultrasound systems were used for the examinations with various dosages of contrast media.

In conclusion, our study results suggest that CEUS is a highly accurate and safe method for classifying focal liver lesions of unclear origin into benign or malignant. Considering its multiple benefits compared to the potential risks associated with ionizing radiation in CT or gadolinium-based contrast agents in MRI, CEUS depicts a promising imaging modality for detecting and characterizing focal hepatic lesions.

## Figures and Tables

**Table 1:** Histopathological results.

Histopathology	Frequency
HCC	38
CCC	4
Metastasis	59
Lymphoma	4
HCC/CCC mixed type	4
Hemangioma	3
Cyst	2
Abscess	3
Hepatitis	4
Fibrosis	10
Cirrhosis	9
Candida infection	1
FNH	2
Hemangioma/FNH mixed type	1
Echinococcus cyst	1
Focal hepatic steatosis	3
Other reactive changes	3
Inconspicuous	13

HCC: hepatocellular carcinoma; CCC: cholangiocellular carcinoma; FNH: focal nodular hyperplasia.

**Table 2:** Distribution positive and negative results from CEUS among malignant and benign focal liver lesions determined by histopathology.

	Total (*n* = 160)	CEUS Positive	CEUS Negative	
				Sensitivity
Malignant	*n* = 109	103	6	94.5%
				Specificity
Benign	*n* = 51	15	36	70.6%
		TPR/ Concordance	TNR/ 1-Discordance	
		87.3%	85.7%	

CEUS: contrast-enhanced ultrasound; TPR: true positive rate; TNR: true negative rate.

**Table 3:** Misclassifications of CEUS in comparison to the histopathological results.

Misclassification	Histopathology
False positive	Fibrotic lesions: *n* = 7
	Cirrhotic lesions: *n* = 3
	No pathologic changes: *n* = 2
	Adenoma: *n* = 1
	Reactive changes: *n* = 1
	Abscess: *n* = 1
False negative	HCC: *n* = 4
	Neuroendocrine tumor (Metastasis): *n* = 1
	Adenoma carcinoma (Metastasis): *n* = 1

HCC: hepatocellular carcinoma.
